# Maternal health care services utilisation in the context of ‘Abiye’ (safe motherhood) programme in Ondo State, Nigeria

**DOI:** 10.1186/s12889-020-08512-z

**Published:** 2020-03-19

**Authors:** Anthony Idowu Ajayi, Wilson Akpan

**Affiliations:** 1 Population Dynamics and Sexual and Reproductive Health and Rights Unit, African Population and Health Research Center, Manga Close, Off Kirawa Road, Kitisuru, Nairobi, 00100 Kenya; 2 Research and Innovation, Walter Sisulu University, Nelson Mandela Drive, Mthatha, South Africa

**Keywords:** “Abiye”, Safe motherhood, Maternal health care utilisation, And skilled birth attendant

## Abstract

**Background:**

The Nigeria Demographic and Health Survey (NDHS) of 2008 show that Ondo State had the worst maternal outcomes in the South Western region of Nigeria. To address this problem, the “Abiye” (safe motherhood) programme—which included community engagement, health system strengthening and user fee removal— was implemented by the state government. We assessed the use of maternal health care services and its determinants at 5 years after the implementation of this programme using a population-based survey. We also compared the results of our survey to the NDHS 2013 to assess improvement in maternal health care services utilisation.

**Methods:**

We conducted a population-based survey in 2016 among representative sample of 409 women who had given birth between 2011 and 2015, which were selected using cluster random sampling. We compared the findings of this 2016 survey to the 2013 NDHS, which contains maternal health care services utilisation information of a total of 434 women who gave birth between 2009 and 2013 to assess progress in the use of maternal health care services. We used descriptive and inferential statistics for our data analysis.

**Results:**

In the 2013 NDHS survey, about 80% of women received antenatal care compared to 98% in the 2016 survey. Our survey shows that the majority of births (85.6%) took place in health facilities compared to only 56.5% in NDHS 2013 survey, which represents a 29.1 percentage points increase. In both surveys, women with primary level of education or less had lower odds of delivering their babies in health facilities. However, while the 2013 NDHS survey shows that women who resided in urban areas were twice more likely to deliver their babies in health facilities compared to those living in rural areas, the 2016 survey shows that urban residence was no longer significantly associated with a higher odds of facility-based child delivery.

**Conclusion:**

Maternal health services utilisation has improved considerably following the implementation of the “Abiye” initiative. The findings of this study suggest that with community engagement, health system strengthening and user fee removal for the most vulnerable, universal access to and utilisation of maternal health services is possible.

## Background

Nigeria has a disproportionately high maternal mortality rate, with about 19% of global maternal deaths [1–5]. With over 50,000 maternal deaths annually, Nigeria is one of the most dangerous countries in which a woman can give birth [3]. There is evidence that most maternal deaths are preventable with access to quality maternal health services; however, most deliveries take place at home and without the assistance of a skilled health worker in Nigeria [6]. According to the 2013 Nigeria Demographic and Health Survey (NDHS), only 36% of births took place in a health facility, and skilled health worker delivered only 38% of all births in the country [6]. It is worth noting that this figure varies by state and place of residence. To address this problem, the federal government has devoted far greater policy attention and resources to maternal health [7], and a handful of state governments are beginning to implement context-specific maternal health interventions [8]. One outstanding example is the “Abiye” (a term that means “safe motherhood”) initiative developed by the Ondo State government in February 2010 [9]. Crafted in response to the NDHS report of 2008, which indicated that Ondo State had the worst maternal outcomes in South-Western Nigeria, the 2010 “Abiye” initiative has been described as the most comprehensive home-grown maternal health intervention programme [10]. The Abiye Programme was initiated to address the four key phases of delay in access to maternal health care services. The four delays are 1) delay on the part of patients to seek care when complication arise, 2) delay in reaching care to poor infrastructure support, communication challenges and transport, 3) delay in accessing care due to poor facilities or no health facilities, 4) delay in referral care for “at risk cases or emergencies”.

Aside from the studies that examined the effects of the programme on maternal health care utilisation during the piloting phase [11–13] and the Ondo state’s evaluation of the programme, no study has reported on the effect of the “Abiye” initiative on the utilisation of maternal health services. The full report of the evaluation of the programme by the state government remained unpublished; however, a snapshot of their findings is available on their website [14]. This study assessed the use of maternal health care services and its determinants at 5 years after the implementation of the “Abiye” programme drawing from a cross-sectional survey. We also compared the results of our study to the NDHS 2013 to assess improvement in maternal health care services utilisation.

### The “Abiye” (safe motherhood) initiative

The overarching objective of the initiative was to improve the utilisation of quality maternal health care with a view to reducing maternal deaths among pregnant women [10]. Specifically, the “Abiye” (safe motherhood) initiative has four strategic components designed to address the four delays that account for poor maternal health in Ondo State [10, 15]. To address the first delay, which is the delay in seeking care, the media warned pregnant women about the dangers of childbirth at home [10, 15]. Furthermore, trained community health workers (CHW), otherwise known as health rangers, had the task of reaching all pregnant women where they lived, worked and played. In order to make their work effective, each ranger had oversight over only 25 pregnant women [10, 15]. Their role included regular communication with the pregnant women, regular visits to them, monitoring them with the use of a customised checklist to determine women who were at risk in pregnancy and to counsel them on family planning and birth preparedness [10, 15]. The pregnant women were provided with mobile phones so they could maintain free contact with their health rangers and health care providers during the pilot programme in one of the local government areas. The second strategic component addressed the second delay, which is the delay in reaching care. New primary health centres were built in underserved communities to improve access and ensure that pregnant women have health facility in close proximity to where they live and old ones were refurbished and equipped with human and material resources.

The third component addressed the third delay, which is the delay in accessing care. Recognising that women delay in seeking care due to financial difficulties, user fee was exempted for all pregnant women in all government clinics and hospitals. This involved massive infrastructural upgrades in all government health facilities in all localities within the State and construction of new facilities where there were no basic health centres. There was also the provision of drugs and necessary consumables to ensure that patients were attended to promptly and properly. Furthermore, hundreds of doctors, nurses and midwives, recruited into the state workforce, received good incentives [10, 15].

The last strategic component addressed the delay in referring care. Also, the Rangers received tricycles, which could serve as ambulances in rural areas, to transfer women to tertiary facilities during emergencies [10, 15]. The government built two state-of-the-art mother and child hospitals to take care of emergencies in the State [10, 15]. To discourage the use of Traditional Birth Attendants (TBAs) and Faith-Based Organization (FBAs), a conditional cash transfer programme was initiated which incentivised TBAs and FBAs for every pregnant woman referred to the clinic. The “Abiye” initiative was summarised by Cooke and Tahir [16] as a health project where all pregnant women in the state were registered and linked to care with mobile phones and the provision of completely free tertiary level maternity care. Bill and Melinda Gates, Department of International Development (DFID), Society for Family Health (SFH), World Health Organisation (WHO) and Ford Foundation were notable partners of the “Abiye” Initiative in Ondo State [10, 16].

The Abiye initiative began with a study that examined the primary reasons why pregnant women did not seek or delay in seeking care. This finding of this study was then used to develop the Abiye Initiative, which began with a pilot programme in one out of the eighteen local government areas of the state. The pilot programmes helped to identify what works and what did not work. For example, the pilot project helped to policymakers to determine that “tricycle as ambulance” for pregnant women needing referrals would be a waste of resources given that it is an inappropriate means of transportation for transporting a pregnant woman in emergency cases in resource-poor settings with poor terrains, lack access road networks. Also, the pilot programme helped the policymakers to desist from giving all pregnant women mobile phones given its use during the piloting.

## Methods

### Study area

The study took place in Ondo State, Nigeria, between May and September 2016. According to the most recent population estimate, around 4,671,700 people were living in the Ondo State in 2016 [17]. The state is located in the Southwestern region of Nigeria and populated mainly by the Yoruba speaking people of Nigeria. Subsistence farming, fishing, and trading are the main occupations of people in the state. The state has a higher proportion of urban dwellers compared to rural dwellers. Ondo State has the worst maternal outcomes in the region, according to the 2013 and 2008 NDHS reports. In 2013, up to 15% of pregnant women did not attend antenatal care, and about 44% of them did not utilise a skilled birth attendant for child delivery [6]. According to the Ondo State Ministry of Health, 40% of women of reproductive age are using modern contraception, and 86% of HIV+ pregnant women are on anti-retroviral therapy [14].

### Study design and sampling

**Fig. 1:**
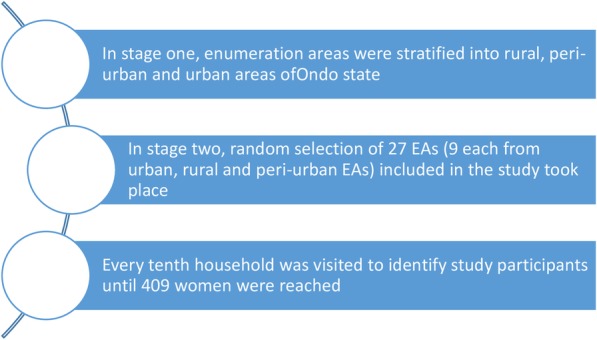
overview of participant selection

This cross-sectional evaluation population survey was conducted between May and September 2016. The current survey is referred to as the endline survey in this study, and the 2013 NDHS is referred to as the baseline survey. The sample size of 409 was estimated using the sample size calculator (http://web1.sph.emory.edu/users/cdckms/samplesize%20icc%20deff2.html), at a confidence coefficient of 95%, a confidence interval width of ±5, 800,000 live births over the 5 years (2011–2015), design effect of 2, 15 observations per enumeration areas and a total of 27 enumeration areas. A two-stage cluster random sampling method facilitated the selection of a representative sample of women included in the study. The state was clustered into enumeration units and stratified based on rural and urban areas. Through simple random sampling, the research staff identified Enumeration Areas (EAs) from the list of EAs in the 2006 census, with probability proportional to size. We selected a minimum of 15 households in each EA until they reached a sample of 409. To accommodate new houses built since 2006, which were not included in EAs, they selected every 10th household in each EA. We skipped households without women who gave birth over the period of study, and we only selected one woman per household, irrespective of the number of eligible women living in the household. The overview of the participants’ selected is presented in Fig. 1.

### Baseline survey

Since there was no baseline survey available, we used the data from the 2013 NDHS as a baseline to enable us to assess improvement in the use of maternal health care services post-implementation of the “Abiye” programme. The 2013 NDHS data is publicly available and could be obtained from Measure DHS (https://www.dhsprogram.com/Data/). The sampling method used in the DHS is similar to that of the current study. We downloaded the women recode data and extracted relevant variables on maternal health care utilisation and socio-demographic characteristics. A total of 432 women in Ondo State selected using 2-stage cluster random sampling were included in the NDHS 2013 survey. Our comparison is possible given that we used the DHS questionnaire for the current study.

### Participants

Participants were women within the reproductive age (15–49 years) that gave birth to at least one child over the 5 years (2011–2015) post-implementation of the “Abiye” programme in Ondo state. Women who did not deliver between 2011 and 2015 were excluded. Women aged 50 years or more were excluded.

### Method of data collection

Trained research assistants, together with the first author, administered the questionnaire to the study participants. The questionnaire consisted of pre-validated questions adapted from the Nigeria Demographic and Health Survey [6]. Before administering the questionnaire, research staff conducted a pilot study among 20 women in a different state.

### Outcome measures

The two main outcome measures in this study are the use of antenatal care and child delivery care services. Use of antenatal care was measured by asking the following questions: *When you got pregnant after your last birth, did you see anyone for prenatal care for this pregnancy? Whom did you see*? *Where did you receive antenatal care for this pregnancy*? Each question had a nominal level response category (yes/no) measure and assessed an important dimension of the use of prenatal care. All participants were asked where they delivered their index pregnancy to assess utilisation of facility-based child delivery,. Responses were categorised as follows: home, traditional birth attendants, faith-based facilities, private health facilities and public health facilities.

### Covariates

The covariates included in the study are; age, place of residence, level of education, religion, ethnic groups, income, employment status, marital status, watching of television, and wealth status. Age was measured as a continuous variable but later categorised into three categories (15–24, 25–34, and 35–49 years). To measure the level of education, two questions were posed: 1) “*Have you ever attended school*?” and, 2) “*What was the highest level of school you attended*?” The first question had a nominal response category (that is, yes or no), while the second question had an ordinal level of measurement (primary, secondary, and higher education) as the response category. To measure employment and income, participants were asked: “*Do you have a job*? *How much is your income monthly*?” A categorical response (yes or no) to the first question would suffice, and an interval measurement summarised the answers to the second question. An income between 1 to 20,000 Naira scored one; two indicated an income above 20,000 Naira. Participants were asked if they watch television, and a ‘yes’ or ‘no’ response category was assigned.

### Socioeconomic status

In this study, ten questions on levels of education, income, employment status, ownership of mobile phones, regular watching of television, use of bank accounts and the Internet were used to measure socioeconomic status. Participants’ socioeconomic status was derived by summing up the scores accrued to each participant from the questions assessing the level of education, employment status, income, ownership of mobile phones, regular watching of television, use of bank accounts and the Internet. A total score of 10 represented the highest socioeconomic status. A score between 0 and 4 was regarded as low socioeconomic status, scores between 5 and 7 as a moderate socioeconomic status, while a score between 8 and 10 reflected high socioeconomic status.

### Statistical analysis

The data analysed here were drawn from a larger study, which examined maternal outcomes in the context of free maternal health care [18]. Obtained data were coded and captured into the Statistical Package for the Social Sciences (SPSS version 24). Mean and frequency distribution of all variables of interest were computed. Chi-square statistics and Fisher’s exact test were used to examine determinants of maternal health care services utilisation. Also, adjusted and unadjusted logistic regression models were computed to examine the determinants of maternal health care services utilisation. Alpha values less than 0.05 were considered to be statistically significant. To account for the complex sampling strategy, sampling weight was applied, and analysis was performed using the complex sample feature of SPSS.

### Ethical considerations

The University of Fort Hare’s Research Ethical Committee (UREC) approved the study protocol (AKP031SAJA01). Furthermore, the Ondo State Health Research Ethics Committee (OSHREC) reviewed and approved the study protocol [NHREC/18/08/2016]. Community leaders and household heads in the study settings granted the researchers permission to conduct the study. Participation was voluntary, and all study participants signed written informed consent. Rights to privacy and anonymity were respected throughout the study. Parent consent and assent were obtained for the inclusion of a few respondents less than 18 years in the DHS data.

## Results

The demographic characteristics of participants in both surveys are presented in Table 1. The average ages of study participants for baseline (NDHS 2013) and endline (current study) were 31.29 (7.28) and 31.7 (SD 6.2) years, respectively. Most of the participants in both surveys were above 24 years, employed, married and Christians.

**Table 1:** Weighted demographic characteristics of study participants

Variables	NDHS 2013 (Baseline survey)		Current study (Endline survey)	
	Frequency	Percent	Frequency	Percent
Education Level				
Primary or less	183	42.2	71	18.2
Secondary	195	44.9	219	54.6
Higher degree	56	12.9	112	27.9
Age				
≤ 24	82	18.9	40	10.0
25–34	196	45.2	226	56.2
35–49	156	35.9	136	33.8
Marital Status				
Ever married	434	100	399	99.2
Never married	–	–	3	0.7
Place of residence				
City	172	39.6	174	43.3
Rural areas	262	60.4	228	56.7
Religion				
Christian	358	82.5	329	81.8
Muslim	76	17.5	73	18.2
Ethnic group				
Yoruba	249	57.4	340	84.6
Others	185	42.6	62	15.4
Employment status				
Employed	370	85.3	350	87.1
Unemployed	64	14.7	52	12.9
Watch television				
Yes	324	74.7	383	95.3
No	110	25.3	19	4.7
Children ever born				
1	89	20.5	91	22.6
2	58	13.4	94	23.4
3	74	17.1	113	28.1
4	72	16.6	69	17.2
5 and above	141	32.5	35	8.7
Number of children between 2011 and 2015				
1	247	56.9	219	54.5
2	164	37.8	161	40.0
3	23	5.3	22	5.4
**Wealth status**				
Poorer	95	21.9		
Middle	110	25.3		
Richer	116	26.7		
Richest	113	26.0		

### Antenatal care utilisation and its determinants before and after the implementation of the “Abiye” initiative in Ondo state

In the 2013 NDHS (baseline survey), about 80% of women received antenatal care compared to close to 98% in the current study (Table 2). While the proportion of women who received antenatal care services varied by age, place of residence, education level, religion, ethnic groups, and wealth status at baseline, these disparities have significantly reduced at post-implementation of the Abiye programme (endline survey), with noticeable improvement across all demographic characteristics.

**Table 2:** Antenatal care utilisation and determinants before and after the implementation of the “Abiye” initiative in Ondo state

Variables	2013 NDHS (Baseline survey)		Current study (Endline Survey)	
	More than one Antenatal care visit n(%)	No antenatal care visit n (%)	More than one Antenatal care visit n(%)	No antenatal care visit n(%)
**All participants**	345 (79.5)	89 (20.5)	393 (97.8)	9 (2.2)
**Age**				
15–24 years	57 (69.5)	25 (30.5)*	38 (95.0)	2 (5.0)
25–34 years	163 (83.2)	33 (16.8)	222 (98.2)	4 (1.8)
35–49 years	125 (80.1)	31 (19.9)	133 (97.8)	3 (2.2)
**Place of residence**				
Urban	168 (97.7)	4 (2.3)***	173 (99.4)	1 (0.6)*
Rural	177 (67.6)	85 (32.4)	220 (96.5)	8 (3.5)
**Levels of education**				
Primary Education or less	114 (62.3)	69 (37.7)***	67 (94.4)	4 (5.6)*
Secondary Education	176 (90.3)	19 (9.7)	214 (97.7)	5 (2.3)
Higher Education	55 (98.2)	1 (1.8)	112 (100.0)	0 (0.0)
**Religion**				
Christians	276 (77.1)	82 (22.9)*	323 (98.2)	6 (1.8)
Muslims	69 (90.8)	7 (9.5)	70 (95.9)	3 (4.1)
**Ethnic groups**				
Yoruba	236 (94.8)	13 (5.2)***	331 (97.4)	9 (2.6)
Others	109 (58.9)	76 (41.1)	62 (100.0)	0 (0.0)
**Watch television regularly**				
Yes	287 (88.6)	37 (11.4)***	374 (97.7)	9 (2.3)
No	58 (52.7)	52 (47.3)	19 (100.0)	0 (0.0)
**Socio-economic status**				
Low socioeconomic status			33 (97.1)	1 (2.9)
Middle income			232 (96.7)	8 (3.3)
High socio-economic status			126 (100.0)	0 (0.0)
**Wealth status**				
Poorer	41 (43.2)	54 (56.8)***		
Middle	90 (81.8)	20 (18.2)		
Richer	105 (90.5)	11 (9.5)		
Richest	109 (96.5)	4 (3.5)		

***P-value< 0.001, *P-value< 0.05, *P*-values calculated from Pearson Chi-Square

### Utilisation of health facilities for childbirth and its determinants before and after the implementation of the “Abiye” initiative

**Fig. 2:**
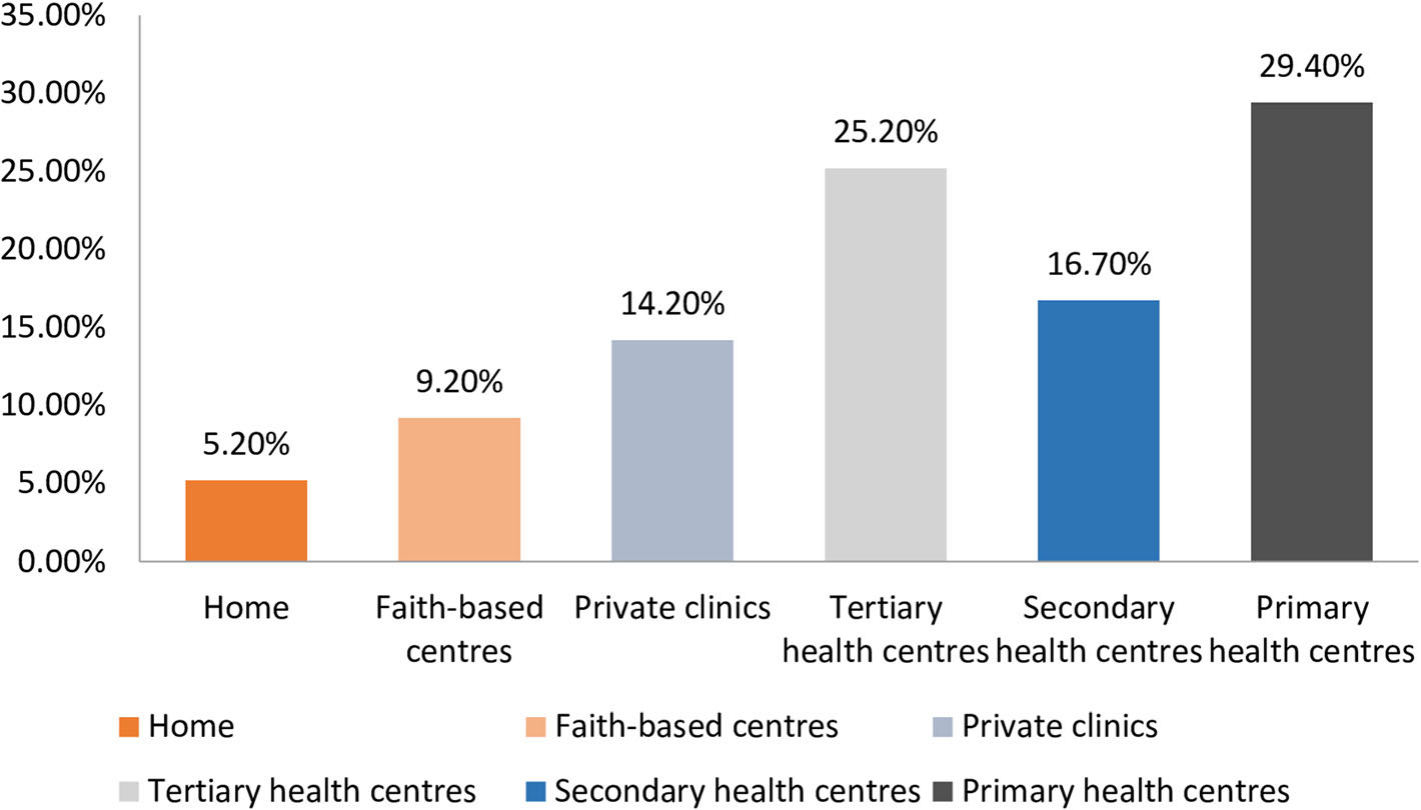
Distribution of participants by place of child delivery

The findings showed a marked improvement in the use of facility-based delivery. The use of facility-based child delivery has increased from 56.5% in NDHS 2013 to 85.6% in 2016 (endline survey) (Table 3). The distribution of births by place of delivery in the endline survey is shown in Fig. 2, with only 5.2% of all births delivered at home, and 71.4% in a government-owned health facility. In both the baseline and the endline surveys, women with primary level of education or less had lower odds of delivering their children in health facilities. However, while women with primary level of education or less in the baseline survey were up to 94% less likely to embrace facility-based delivery, the odds of facility-based child delivery has reduced to 74% lesser likelihood in the endline study. Also, while women who resided in urban areas in the baseline survey were twice more likely to deliver their children in health facilities compared to those living in rural areas, the current survey shows that rural and urban disparity in the use of facility-based child delivery has disappeared. Likewise, disparities in use of facility-based child delivery by ethnic groups, religion, and watching of Television, which were pronounced in the baseline survey, have declined to negligible levels as indicated in Table 3.

**Table 3:** Adjusted logistic regression showing determinants of facility-based childbirth in current study compared to NDHS 2013

Variables	2013 NDHS (Baseline survey)			Current study (Endline survey)		
	%	UOR	AOR	%	UOR	AOR
**All Participants**	245 (56.5)			344 (85.6)		
**Age**						
15–24	42 (51.2)	0.66 (0.38–1.13)	0.77 (0.40–1.51)	34 (85.0)	1.34 (0.51–3.52)	0.96 (0.34–2.74)
25–34	107 (54.6)	0.75 (0.49–1.15)	0.64 (0.38–1.07)	200 (88.5)	1.82 (1.01–3.28)	1.60 (0.86–2.97)
35–49	96 (61.5)	1	1	110 (80.9)	1	1
**Level of education**						
Primary Education or less	68 (37.2)	0.03 (0.01–0.11)***	0.06 (0.02–0.20)***	56 (78.9)	0.33 (0.13–0.79)*	0.24 (0.09–0.62)*
Secondary Education	124 (63.6)	0.10 (0.03–0.33)***	0.11 (03–0.37)***	185 (84.5)	0.48 (0.22–1.03)	0.39 (0.18–0.88)*
Higher Education (ref)	53 (94.6)	1	1	103 (92.0)	1	1
**Place of residence**						
Urban	132 (76.7)	4.35 (2.83–6.60)***	2.10 (1.24–3.56)*	145 (83.3)	0.73 (0.42–1.27)	0.61 (0.33–1.13)
Rural	113 (43.1)	1	1	199 (87.3)	1	1
**Ethnic groupings**						
Yoruba	176 (70.7)	4.05 (2.71–6.07)***	1.73 (1.04–2.86)	291 (85.6)	1.01 (0.47–2.18)	0.73 (0.33–1.63)
Others	69 (37.3)	1	1	53 (85.5)	1	1
**Religion**						
Christian	181 (50.6)	0.19 (0.10–0.37)	0.16 (0.08–0.32)***	277 (84.2)	0.92 (0.83–1.01)	0.44 (0.17–1.11)
Islam	64 (84.2)	1	1	67 (91.8)	1	1
**Watch television**						
Yes	212 (65.4)	4.42 (2.77–7.05)	1.92 (1.10–3.35)*	328 (85.6)	1.12 (0.32–3.97)	0.85 (0.23–3.17)
No	33 (30.0)	1		16 (84.2)	1	1

*AOR* adjusted Odds Regression, *UOR* Unadjusted Odds regression, ***P value< 0.001, *P value< 0.05.

## Discussions

This study examined the effect of the “Abiye” initiative on the utilisation of skilled birth facilities in Ondo State, Nigeria. We found that almost all women utilised antenatal care services during their index pregnancy in the 2016 survey, which suggests an improvement compared to previous estimates of antenatal care utilisation in the 2013 NDHS [6]. The findings also indicated that facility-based child delivery has increased from 56.5% (baseline survey) to 85% (endline survey), which is a 29-percentage points increase in child delivery in health facilities across the state. The finding of this study is consistent with the results of an evaluation study conducted by the state government [10] and the latest 2018 NDHS recently released [19]. Also, our findings are consistent with previous studies that have established that user fee removal for maternal health care services is linked with increased use of maternal health care services [20–26].

However, there is still a variation in the proportion of women that utilise facility-based child delivery by the level of education, which indicates that use of facility-based child delivery is not universal. Despite the impressive results, some women with lower level of education appear to still favour faith-based childbirth. This suggests that the ‘Abiye’ initiative is has not adequately discouraged the utilisation of faith-based services. This is finding is consistent with the fact that scholars agree that user fee exemption policy is effective, but alone is not sufficient to eliminate inequality of access to maternal health care services’ utilisation [27, 28]. As such, a new approach is required to discourage the use of faith-based services, especially in urban areas among less educated women. This will require a better understanding of the reasons why pregnant women prefer faith-based services.

Nevertheless, in the case of Ondo State, the programme implemented is comprehensive and the improvement observable after the implementation of the “Abiye” programme is drastic and commendable. As demonstrated by the “Abiye” intervention, improving access to maternal health services requires a better understanding of the contextual problems and implementation of several approaches tailored to addressing those peculiar problems. As is the case in Ondo State, in areas where there is no health facility, building one is a better policy compared to user fee removal only. However, it is must be noted that the cost required to build new health facilities and the infrastructures is enormous and given the paucity of resources in many sub-Saharan African (SSA) setting, this is difficult, if not impossible, to implement across all settings lacking health facilities in SSA.

Another thing that works in the case of the “Abiye programme” was the partnership with the rulers to forbid child delivery at home in settings where it is customary for women to give birth at home as well as the use of lone rangers to visit and educate pregnant women. In the case of Ondo State, a comprehensive evidence-based intervention programme was implemented, which included health system strengthening, infrastructure upgrades, construction of new facilities, recruitment of new health workers, the training of health workers, user fee removal, incentivising of traditional birth attendants to refer pregnant women to health facilities and partnership with and support from community leaders [16]. The impressive results of the “Abiye” initiative are a clear indication that universal access to maternal health care services is possible with leaders committed to this goal and also with the implementation of a comprehensive evidence-based programme rather than one stand alone intervention [15]. The sustainable development goal’s overarching principle is leaving no one behind. The example of Ondo State demonstrates that it is possible to improve the utilisation of maternal health care services if there is political will. The “Abiye” programme is evidence-based and the elements of the programme have been demonstrated to be effective in many different settings [10, 15]. The programme has received mostly positive reviews from beneficiaries [29].

### Study strengths and limitations

Although this study provides insights into the effects of the“Abiye” initiative on the maternal health care services’ utilisation, the findings must be understood in the context of the limitations. This study did not use an experimental design, and there are plethoral of programmes implemented concurrently, as such it is difficult to assess what in particular is the most effective intervention. However, our use of cross-sectional design, variable measurement and sampling methods allow us to estimate use of maternal health care services and its determinants and compared our findings with 2013 NDHS results. The fact that our findings are consistent with NDHS 2018 [19] and the post-implementation evaluation survey findings but the Ondo State Ministry of Health is a strength of this study.

## Conclusion

Maternal health services’ utilisation has considerably improved following the implementation of the “Abiye” (safe motherhood) initiative. The findings of this study suggest that, with community engagement, health system strengthening and user fee removal for the most vulnerable, universal access and utilisation of maternal health services is possible. Despite the impressive results, some women appear to favour faith-based childbirth suggesting that the “Abiye” (safe motherhood) initiative is not sufficient to discourage the utilisation of faith-based services. Concerted efforts should be concentrated towards sustaining this programme and also addressing the preference of faith-based child delivery by less educated women.

## Data Availability

The datasets generated and analysed during the present study are available from the corresponding author on reasonable request.

## Abbreviations

EA: Enumeration Area
FBA: Faith-based attendant
NDHS: Nigeria demographic and health survey
SFH: Society for Family Health
WHO: World Health Organisation
